# Personality Prototypes in People with Type 1 Diabetes and Their Relationship with Adherence

**DOI:** 10.3390/ijerph18094818

**Published:** 2021-04-30

**Authors:** Carmen Sánchez-Urbano, María J. Pino, Carlos Herruzo

**Affiliations:** Psychology Department, Facultad de Ciencias de la Educación, University of Cordoba, 14071 Cordoba, Spain; carmensanchezps@hotmail.com (C.S.-U.); mjpino@uco.es (M.J.P.)

**Keywords:** personality prototypes, adherence, type 1 diabetes

## Abstract

Type 1 diabetes (Dm1) is a chronic endocrine and metabolic disease that affects the whole person and requires active, decisive treatment. However, personality traits may influence a patient’s adherence to treatment guidelines. The objective of this work is firstly to identify the 3 Asendorpf personality prototypes (resilient, undercontrolled and overcontrolled) in a sample of Dm1 individuals and determine whether there are any differences in comparison with a control sample; and, secondly, to study their association with adherence to self-care guidelines using both physiological indicators (HbA1C) and self-report measures. To achieve these objectives, a descriptive cross-sectional study was carried out. The sample comprised 294 participants, of whom 104 were people with Dm1 and 190 were controls. The participants, aged between 14 and 34 years, were classified by their scores in NEO-FFI-R, according to the personality characteristics inherent to Asendorpf’s prototypes. Asendorpf’s 3 prototypical personality patterns were found both in the group of people with Dm1 and in the control sample. These patterns showed different degrees of association with adherence to self-care guidelines for this disease and with psychological health factors. Importance should therefore be attached to the personality traits and Asendorpf prototypes of people with Dm1 when proposing interventions to address medical, psychological, and behavioral aspects.

## 1. Introduction

Type 1 Diabetes (Dm1) is a chronic metabolic condition caused by an absolute insulin deficiency [1] brought about by the autoimmune destruction of pancreatic β-cells. Treatment includes behavioral and metabolic self-care measures [2] like proper nutrition, avoidance of toxic habits such as tobacco and alcohol consumption, and regular physical exercise [3]. It also includes insulin administration, self-monitoring of blood glucose, and the detection and treatment of hypoglycemia [4]. Dm1 requires a continuum of care with coordinated multifactorial strategies aimed at obtaining adequate health outcomes and addressing such aspects as patient empowerment and education, psychosocial counseling, and the taking into account of community aspects that influence lifestyles, among other things [2].

The diagnosis of Dm1 has a significant impact on the individual, since the prognosis, the medium and long-term complications and the characteristics of the treatment make the patient’s participation indispensable [4] (up to 95% of the disease management responsibilities fall on the patient [5]). Some aspects of Dm1, like its unexpected onset at a young age, the danger of hyper- and hypoglycemia (excessively high or low glucose levels), the complications that may arise (retinopathy, nephropathy, neuropathy…), and the burden of self-care that it entails [6], are justifiably stressful in emotional terms. Adherence to self-care guidelines, defined as a person’s active, voluntary behavior aimed at improving, maintaining, and preventing health deterioration, is therefore essential and decisive in Dm1 outcomes [7]. The main dimensions of adherence can be said to be the patient’s own behavior and their perception of burden or benefit [8]. This is an important aspect to be take into account, because inadequate adherence may result in long-term Dm1 complications [5].

According to some studies [6], Dm1 receives special consideration in the field of health psychology [9] precisely because it is so highly demanding, both psychologically and behaviorally [5]. In this regard, psychological factors would appear to play an important role in Dm1 therapy, since this is an illness that affects people’s lifestyles at early ages. Dm1 requires continuous, complex treatment with no immediate consequences, and self-control over personal behavior is crucial in order to avoid its negative long term effects and optimize prognosis [9].

The role of personality factors has been studied as one of the psychological aspects of this disease [10], and much evidence can be found in the scientific literature regarding their influence on self-care and Dm1 outcomes. In general, personality traits influence health- and illness-related behaviors and are associated with physical health [11,12,13,14]. Some studies have also specifically indicated the influence of psychological factors, both emotional and behavioral, in Dm1, highlighting the very important role they play in adherence to treatment [6,10,15].

In this regard, theoretical models like the Type A Personality Pattern (impatience, competitiveness) and the Type D Personality Pattern (negative affectivity and social inhibition) have been linked with major health issues such as cardiovascular problems [16,17] and low adherence to treatment in Dm2 [18]. Nevertheless, it seems that here Costa and McCrae’s Big Five Model [19] may be particularly relevant since it considers a large number of personality factors and aspects. Indeed, it has been found that each trait plays a role in glycemic control [20] and therefore has a different impact on adaptation, quality of life, and perception of physical and psychological health [21]. Specifically, high Conscientiousness and Agreeableness positively influence glycemic control [20], while low Extroversion and high Openness are associated with adequate personal control [21]. On the other hand, Extraversion and Agreeableness correlate with adherence; and Conscientiousness is related to adherence and health maintenance [7]. Traits that negatively influence adherence are a low level of Conscientiousness and high levels of Extraversion [22] and Neuroticism [23]. High Neuroticism and low Conscientiousness are related to negative long-term consequences of the disease [21].

Based on these five traits, Asendorpf et al. [24], established that the different patterns of personality description are organized into three prototypic patterns, each with their own characteristics: resilient, overcontrolled and undercontrolled. The resilient type is characterized by a reduced level of Neuroticism and a high level of Conscientiousness. It presents flexible responses to changing and stressful situations, which favors good adjustment. The overcontrolled type is related to low Extraversion and high Neuroticism. This type presents poor adjustment and tendencies towards feelings of inhibition, shyness, low self-esteem, or loneliness. The undercontrolled type is related to low Conscientiousness and low Agreeableness. It presents poor adjustment and tendencies to social problems such as lack of self-esteem or antisocial behaviors [24]. The influence of these prototypes on different physical and psychological health problems has been studied. Specifically, their impact has been assessed on subjective health perception [25] and on cardiovascular health outcomes [26]. They have been considered as indicators of adaptation and psychosocial adjustment in adolescents [27] and also as predictors of behavioral problems in children and adolescents [28]. Moreover, they have been related to increased risk of eating disorders [29] and have been used to estimate admission to a rehabilitation program for patients with spinal cord injuries [30].

Given that adherence to self-care guidelines is key to optimizing the progression of Dm1 and avoiding complications derived from it [7], and considering the influence of personality on the perception of and ability to cope with situations in general [12] and with Dm1 in particular [31], it is clearly relevant to take into account the personality characteristics of a person with Dm1 when dealing with the problem of adherence to self-care guidelines for such a demanding illness. Bearing in mind that the Asendorpf prototypes have predicted individuals’ perception of health and risk of presenting different psychological problems/psychosocial adjustments and their implications, and that those same prototypes have been useful in assessing the effectiveness of rehabilitation interventions, the purpose of this work is therefore to describe the personality characteristics of a group of people with Dm1, to establish whether the three Asendorpf prototypes are present, and to determine whether the Dm1 sample differs from a control sample of people without Dm1. These questions have not been addressed in the scientific literature. This paper also assesses the relationship between the Asendorpf prototypes and psychological health factors and other metabolic and behavioral self-care aspects of Dm1. Here, self-care aspects mainly refer to the monitoring of glucose levels, insulin administration, glycosylated hemoglobin (HbA1C) levels and compliance with certain behavioral guidelines (control of toxic habits, frequent physical exercise, suitable diet).

In summary, the objectives of this study are, firstly, to try to identify the three Asendorpf personality prototypes in a sample of people with Dm1 and determine whether there are differences with respect to a control sample; and, secondly, to study their association with adherence to self-care guidelines using physiological indicators (HbA1C), self-report measures, and psychological health variables. We hypothesize that the three Asendorpf prototypes of personality will be found and that people with resilient personalities will show emotional stability and, probably, adequate adherence. We also expect people with undercontrolled and overcontrolled personality prototypes to present greater emotional instability (associated with social and behavioral problems, respectively) and worse adherence to self-care guidelines [29].

## 2. Materials and Methods

### 2.1. Participants

This was a descriptive cross-sectional study. The sample comprised 294 Spanish participants: a total of 104 people diagnosed with Dm1 and a control group of 190. All participants had medium or medium-high levels of education. Regarding sex, 45.60% were men and 54.40% were women (X^2^ = 3.463, *p* = 0.063). The age of the participants was between 14 and 34 years, 47.60% of them being ≤ 21(19.20 ± 2.04 years) and 52.40% of them being older than 22 (25.82 ± 4.11) (X^2^ = 3.377, *p* = 0.066).

### 2.2. Instruments

The information was collected by a paper and pencil survey comprising a general anamnesis and several questionnaires. The anamnesis collected data on age and sex; anthropometric characteristics [height (m) and weight (kg)] for the subsequent calculation of the Body Mass Index (BMI) (kg/m^2^); and aspects related to behavioral habits (consumption of alcohol (number of SDUs) and tobacco (number of cigarettes), physical exercise (frequency per week) and estimation of diet adherence (deficient/regular/adequate)). In addition, the following Dm1-related data were collected: HbA1c levels, times of glucose monitoring, times of insulin administration, years with Dm1 diagnosis and estimation of knowledge about Dm1 (Good/Normal/Poor). Glucose levels are monitored by means of a self-applied technique based on using different devices (glucometers) to determine the current levels of glucose in a person’s blood. This is necessary because food, medication, physical exercise, and stress affect the blood sugar level, and it is essential to know this level before administering the appropriate dose of insulin. People with Dm1 are educated to determine the appropriate dosage and self-administer it through diabetes education services provided mainly by health services.

The questionnaires used were as follows: for personality traits, the NEO-FFI-R (abbreviated version of the NEO-PI-R) was used, consisting of 60 items with five response options. This questionnaire assesses the five major personality traits: Neuroticism (N), Extraversion (E), Openness to Experience (O), Agreeableness (A) and Conscientiousness (C) [19]. It has adequate test-retest reliability, good stability coefficients (0.68–0.83), and good convergent validity indices, which correlate with analogous constructs [16].

The Clinical Outcomes in Routine Evaluation-Outcome Measure (CORE-OM) questionnaire was used for psychological health factors. CORE-OM has 34 items with five response options [32], grouped into four scales: subjective distress (W) (four items), daily functioning (F) (12 items), problems/symptoms (P) (12 items) and risk (R) (six items). In turn, each scale has several subscales. The P scale comprises four subscales, which assess anxiety problems/symptoms (four items), depression problems/symptoms (four items), physical symptoms (two items) and trauma (two items). The F scale comprises three subscales, assessing level of functioning (four items), social relationships (four items) and intimate relationships (four items). The R scale comprises two subscales, assessing self-harm (four items) and harm to others (two items). CORE-OM also provides a total score, grouping together the W, P and F scales; and another total score that includes all four scales (W, P, F and R). These totals indicate the general emotional state of the subject. This questionnaire has high internal consistency (Cronbach’s α 0.75–0.90 for all dimensions) and high test-retest correlation (0.87–0.91).

The information collection dossier also included an information sheet on the study, requesting the collaboration and informed consent of the participants. The dossier for the control group was the same as that of the case sample, except for the absence of data related to the disease.

### 2.3. Procedure

The research project was presented to the Research Ethics Committee of the Andalusian Regional Government Health Services and obtained a favorable report (Act No. 259, ref. 3292, 1-16-2017).

The sample was obtained from a clinical context, namely one of the Endocrinology outpatient clinics of the Reina Sofía University Hospital in Cordoba. Participants were asked to take part in the study with the collaboration of the clinic staff, endocrinologists, and nurses. They were given the information collection dossier, which they completed and then returned to the same office. The control sample was drawn mainly from an academic context and was made up of people who met the stipulated requirements: i.e., they had to be between 14 and 34 years of age and not to have been diagnosed with Dm1.

Once the dossiers had been collected and the different questionnaires corrected, a database was created for subsequent statistical analysis.

### 2.4. Statistical Analyses

First, a descriptive analysis was performed to obtain the mean ± standard deviation of the variables analyzed; likewise, the percentages of the options in the dichotomous variables were also calculated.

For the *adherence* variable in Dm1, actual adherence and perceived adherence were measured. Actual adherence was based on HbA1C levels, a physiological reference parameter for correct self-care [33]. Values were differentiated according to whether they were ≥7%, which is indicative of poor metabolic control of Dm1, or <7%, which would indicate good metabolic control of the disease. Perceived adherence would be formed by the participants’ self-reported data about their tobacco and alcohol consumption, adherence to a suitable diet, and frequency of physical exercise. The difference between the scores of these variables (actual adherence-perceived adherence) was taken as the *adherence estimation error,* thus differentiating between those who overestimated adherence (reporting adequate adherence behavior patterns, when the physiological value indicated the opposite), those who underestimated their adherence (having physiological values indicative of good management but reporting worse self-care patterns), and those whose perception of their self-care patterns was adequate. The direct scores of the five scales in the NEO-FFI-R questionnaire were transformed into centile scores to group them according to Asendorpf’s criteria [21], thus forming three groups. The resilient group included subjects with Neuroticism scores <40 and Conscientiousness (C) scores >60. The overcontrolled group included subjects with Neuroticism scores > 60 and Extraversion scores <40. The undercontrolled group included subjects with Agreeableness scores <40 and Conscientiousness scores <40.

Finally, the differences between prototypes in aspects related to Dm1 and psychological health factors were evaluated using the chi-square test (X_2_) and analysis of variance (ANOVA) for qualitative and quantitative variables, respectively. The relationship between prototypes and psychological health factors was also assessed, both in the case group and in the control group, in order to check the differences that may exist in the relationships between personality tendencies and psychological factors.

## 3. Results

The different Asendorpf prototypic patterns appeared in both the group of people with Dm1 and the control group, but no significant differences were found between the two groups (X^2^ = 6.268, *p* = 0.099). The percentages are shown in Table 1.

With respect to actual adherence, measured by HbA1c, no significant differences were found in the ANOVA between the three prototypic personality patterns. There were differences, however, in perceived adherence (F = 3.664, *p* = 0.033), with resilient personality types presenting greater perceived adherence: that is to say, they were the ones who reported having adequate habits (no excessive consumption of alcohol or tobacco), following a correct diet, and taking frequent physical exercise.

As indicated in the analysis section, a new variable called adherence estimation error was calculated, comparing the results of perceived adherence with those of actual adherence (i.e., the HbA1C value, which is an indicator of the existence of poor or good metabolic control). The ANOVA showed significant differences (F = 4.985, *p* = 0.012) between prototypes, with resilient personality types making more errors in estimating their adherence. It was also the resilient types that reported a greater amount of diabetes education received (F = 12.224, *p* = 0.002). On the other hand, it was the overcontrolled personality types that made the fewest errors in their estimation of adherence, with lower HbA1C levels than the resilient types, and which reported not having received diabetes education. These data are shown in Table 2.

Regarding psychological health factors (measured by the CORE-OM questionnaire), the results indicated that there are significant differences between prototypes in some scales, both in the group of people with Dm1 and in the control group. The values for the different scales (mean ± standard deviation) are shown in Table 3.

With respect to psychological health factors, overcontrolled personality types presented higher scores in psychological problems or discomfort, while resilient types presented the lowest scores (Total CORE [F = 5.227, *p* = 0.009]). This pattern of resilient (highest scores) and overcontrolled (lowest scores) was repeated in the control sample (Total CORE [F = 4.053, *p* = 0.021]).

With respect to problems and symptoms, both in the CORE P scale and in the other subscales, the overcontrolled types had the highest scores, and the resilient types the lowest scores (CORE P [F = 7.354, *p* = 0.002]; CORE P _Anxiety_ [F = 3.508, *p* = 0.038]; CORE P _Depressed_ [F = 9.628, *p* = 0.000]; CORE _Trauma_ [F = 3.998, *p* = 0.025]; CORE P _Physical Symptoms_ [F = 3.194, *p* = 0.050]). In the control group we found the same pattern in the general scale and in the anxiety and depression subscales (CORE P [F = 12.095, *p* = 0.000]; CORE P _Anxiety_ [F = 10.972, *p* = 0.000]); CORE P _Depressed_ [F = 11.864, *p* = 0.000]). There were no significant differences between prototypes in the CORE P _Trauma_ subscale. In the CORE P _Physical Symptoms_ subscale, the undercontrolled personality types had the highest scores and the resilient types had the lowest scores (F = 6.567, *p* = 0.002).

Regarding daily functioning (CORE F), an opposite pattern was observed in relation to the intimate relationships (CORE F _Close_) and functioning level (CORE F _General_) scales. Resilient personality types scored the highest in problems with intimate relationships, and overcontrolled types scored the lowest (CORE F _Close_ (F = 5.925, *p* = 0.005). In daily functioning, however, the overcontrolled types had the highest level of problems and the resilient types had the lowest (CORE F _General_ (F = 7.389, *p* = 0.002). This pattern was repeated in the control group in both the CORE F _Close_ (F = 7.600, *p* = 0.001) and CORE F_General_ (F = 5.293, *p* = 0.007) scales.

Regarding the risk scale, overcontrolled personality types hade the highest scores in all three scales: general (CORE R (F = 7.042, *p* = 0.002)), self-harm (CORE R _Self_ (F = 6.422, *p* = 0.004)), and aggression to others (CORE R _Others_ (F = 9.670, *p* = 0.000)); while resilient types had the lowest scores. The same pattern was seen in the control group as in the people with Dm1, (F = 3.381, *p* = 0.038), with overcontrolled types scoring higher and resilient types scoring lower. In the CORE R scale and the CORE R _Others_ subscale, it was the undercontrolled types that scored higher and the resilient types that scored lower (CORE R (F = 3.284, *p* = 0.042) and CORE R _Others_ (F = 3.319, *p* = 0.048)).

## 4. Discussion

In accordance with the first aim of this study, we identified the three prototypical Asendorpf personality patterns in a sample of people with Dm1 and in a control sample. These patterns showed different degrees of association with adherence to self-care guidelines for Dm1 and with psychological health factors, once again confirming the existence of these personality patterns that are differentially related to psychological phenomena [24,29]

According to the results obtained in the present study, there are incongruences between actual adherence (HbA1C levels) and estimated adherence (self-reported self-care behaviors) in people with Dm1. These findings suggest a need to monitor adherence to self-care behaviors at a level beyond the individual’s perception [3]. People with Dm1 evidently make errors when estimating their adherence, with significant differences being observed between Asendorpf personality prototypes. The patient’s perception of adherence includes elements like attitudes, perception of benefit or burden of treatment, appreciation, and commitment [8], aspects that involve individual differences consistent with the differences we obtained with regard to personality traits.

Resilient people make more errors in estimating adherence: they report good perceived adherence that is not in accordance with the elevated HbA1C levels they actually have, thus, showing overconfidence. This kind of perception error is congruent with the good adjustment usually shown by resilient people, because personality influences how people perceive and cope with stressful situations [28], and high HbA1C levels are mostly not related to emotional distress [34]. Some studies, however, indicate a low interaction between adherence levels and personality styles [35], and link traits associated with the resilient prototype (low neuroticism and high conscientiousness [24]) to adequate adaptation and adherence [12,21,22]. Ultimately, resilient people with Dm1 may exhibit adequate adaptation, but not adequate adherence, suggesting a positive self-presentation bias. This is also compatible with social desirability, a feature which, as suggested in the literature [36], may be associated with resilient personality.

On the other hand, it is the overcontrolled personality types that make the fewest errors in the estimation of their adherence and show lower levels of HbA1C—a physiological indicator of correct self-management. However, this adequate metabolic state of Dm1 does not translate into psychological well-being, since those same personality types are the ones that present more emotional, daily functioning and risk problems. The characteristics of this prototype, high neuroticism and low extroversion [24], can be related to beliefs about Dm1 control and perceived consequences which lead to low emotional self-esteem [37].

It seems clear that personality factors have an influence on how people cope with and adapt to the Dm1 situation [21]. According to the American Diabetes Association, effective behavioral management and adequate psychological self-esteem are fundamental to achieve treatment goals [1]. And indeed, individuals in the sample evaluated in this study, belonging to different prototypes, were affected differently by psychological health factors. In general, overcontrolled types were related to a higher level of problems or emotional distress and risk, while resilient types showed the least problems in this area. This pattern was repeated in the control sample.

Diabetes is a challenging situation which impacts a person’s lifestyle and requires adaptation to self-care demands [31]. More specifically, having diabetes has been associated with an increased level of anxiety or distress [31] and an increased risk of depression [38]. But although overcontrollers may be associated with higher levels of anxiety and/or depression problems [24], our results suggest that personality type does not have a negative impact on adherence, as indicated in other studies [34,37,39]. On the other hand, resilient people scored the lowest in emotional problems and/or symptoms. This coincides with the fact that resilience influences the number, intensity, and duration of stress symptoms [40]. Although we found that resilient people had the highest HbA1C levels, this ran contrary to studies indicating that elevated HbA1C levels are related to anxiety [41] and psychological stress [42,43], although other studies have also indicated that elevated HbA1C levels are mostly not related to the presence of stress [34].

Regarding daily functioning, an important aspect due to the influence of Dm1 on lifestyle, resilient people were the least affected in their level of daily functioning but they presented more problems in the area of functioning in close relationships, i.e., they showed a tendency to feel loneliness and lack of support and affection. This is probably explained by social desirability in the answers to the questionnaires, as has been observed in a study into the resilient prototype [36]. This condition, together with high critical awareness, is associated with the high Conscientiousness scores [19] typical of the resilient prototype, and may bring about a greater sense of loneliness, isolation, or lack of support [32]—aspects that are included in the functioning in intimate relationships subscale of the CORE-OM. These results were repeated in the control participants, and this may be indicative of the presence of a response style in resilient individuals [36]. On the other hand, it was the overcontrolled personality type that reported fewer problems in functioning in close relationships, although this type also showed greater problems at the level of daily functioning, mediated by emotional problems and feelings of inhibition and shyness [24]. Thus, although Dm1 does not increase the risk of psychosocial problems [44], social support favors health outcomes, is a buffer for stressful events, and is related to greater adjustment to illness [45].

Finally, with respect to risk dimension, overcontrolled people were the ones who scored highest in this area, both in self-harm and aggression to others. This may be related to characteristics of this prototype such as low social self-esteem, lower friendship contacts or inhibition and shyness [24]. In general, Dm1 is associated with a higher presence of suicidal ideation [46]; and suicidal thoughts have been related to non-adherence to treatment [47]. However, this does not coincide with our results, since the overcontrolled people in this study presented adequate adherence, as evidenced by low HbA1C levels. Nevertheless, there may be an interaction between physical and psychosocial factors affecting quality of life and this can probably lead to problems of adjustment to illness in overcontrolled people with Dm1 [6]. This group obtained the worst scores in physical symptoms and the other subscales of psychological problems. On the other hand, the resilient prototype seemed to be indicative of good adjustment, as resilient people scored the lowest in this dimension.

It would be interesting to be consider the variables studied in this paper in order to improve adherence to self-care guidelines and prevent poor evolution of Dm1. Many studies indicate that therapeutic protocols for this disease should include physical, psychological, and social aspects [48], and that behavioral interventions could promote medical and psychological outcomes in Dm1 [15]. Interventions for psychological problems like depression can improve adherence [49]. An early assessment of psychological comorbidity and psychosocial counseling would therefore be desirable to prevent problems and improve Dm1 evolution. This is supported by data indicating that decreases in adherence to treatment could reflect problems in decision making and executive function [50]; that intervention in psychological problems such as depression improves health outcomes in Dm1 [51] and adherence [49]; and that multicomponent interventions and operant procedures would probably be effective in improving adherence, with the cognitive-behavioral strategy looking particularly promising [52].

However, all these issues must be considered with caution. Our work is a cross-sectional study. It would be necessary to assess longitudinally whether changes occur in these personality traits over the years of diagnosis and therefore whether such traits have any other type of influence on adherence. Our sample of cases was made up of a small number of participants, so the number of people belonging to each prototype was also small. Furthermore, the questionnaires used were self-reported, and there may have been biases in the responses, which could have been influenced by different factors (time to answer, context of completion, emotional state in the context of a medical check-up…).

## 5. Conclusions

The different Asendorpf prototypes appear in people with Dm1 and have been shown to relate to aspects of adherence to self-care guidelines. Resilient individuals with objective data showing poor self-management (high HbA1C levels) are those with the greatest error in adherence estimation, while overcontrolled types with HbA1C levels indicative of adequate metabolic control are those with the lowest error in adherence estimation. With respect to psychological health factors, the resilient prototype is associated with good adjustment, with fewer emotional problems and with less risk, whereas overcontrolled personalities are more affected by emotional problems, daily functioning, and risk.

It would be important to consider the personality traits and the Asendorpf prototypes of people with Dm1 in order to propose interventions that take into account medical, psychological, and behavioral aspects, because this could tell us in advance who would be more compliant with self-care recommendations (overcontrolled personalities) and who would be more optimistic in their estimation of their adherence, both of which factors could later influence Dm1 outcomes and prognoses.

## Figures and Tables

**Table 1:** Prototype percentages in Dm1 and Control samples.

Group	Not Classified (%)	Resilient (%)	Overcontrolled (%)	Undercontrolled (%)
Dm1	51.90	24.00	6.70	17.30
Control	50.00	14.70	12.10	23.20

X^2^ = 6.268, *p* = 0.099.

**Table 2:** Adherence data.

Adherence Data	Resilient	Overcontrolled	Undercontrolled	F	*p*	ŋ^2^
HbA1c	7.62 ± 1.16	6.68 ± 1.43	7.25 ± 0.98	1.484	0.240	
Perceived Adherence	0.99 ± 0.90	1.61 ± 0.87	1.74 ± 0.97	3.664	0.033	0.137
Estimation error	1.70 ± 1.45	−0.32 ± 1.74	0.42 ± 1.54	4.985	0.012	0.222

HbA1C = Glycosylated hemoglobin.

**Table 3:** CORE-OM questionnaire scores by Asendorpf personality prototype patterns.

Scale	Group	Resilient	Overcontrolled	Undercontrolled	F	*p*	ŋ^2^
Total CORE	Dm	1.12 ± 0.25	1.73 ± 0.80	1.36 ± 0.44	5.227	0.009	0.196
	C	1.28 ± 0.36	1.58 ± 0.41	1.41 ± 0.39	4.053	0.021	0.082
CORE W	Dm	1.93 ± 0.34	2.46 ± 0.83	1.88 ± 0.77	2.545	0.090	0.102
	C	2.21 ± 0.67	1.90 ± 0.52	2.07 ± 0.64	1.496	0.230	
CORE P	Dm	0.73 ± 0.49	1.68 ± 0.86	1.24 ± 0.70	7.354	0.002	0.246
	C	0.92 ± 0.54	1.82 ± 0.73	1.21 ± 0.69	12.095	0.000	0.210
CORE P_Anxiety_	Dm	0.92 ± 0.69	1.71 ± 0.88	1.32 ± 0.79	3.508	0.038	0.135
	C	0.90 ± 0.59	1.87 ± 0.71	1.31 ± 0.83	10.972	0.000	0.194
CORE P_Depressed_	Dm	0.47 ± 0.57	1.64 ± 0.96	0.99 ± 0.62	9.628	0.000	0.300
	C	0.73 ± 0.55	1.76 ± 1.08	1.01 ± 0.70	11.864	0.000	0.207
CORE P_Trauma_	Dm	1.00 ± 0.77	2.00 ± 0.82	1.59 ± 1.15	3.998	0.025	0.151
	C	1.38 ± 1.04	1.85 ± 0.79	1.42 ± 0.96	1.951	0.148	
CORE P_P.S._	Dm	0.63 ± 0.61	1.36 ± 1.07	1.12 ± 0.91	3.194	0.050	0.124
	C	0.89 ± 0.79	1.80 ± 0.91	1.16 ± 0.99	6.567	0.002	0.126
CORE F	Dm	1.84 ± 0.32	2.04 ± 0.51	1.72 ± 0.55	1.267	0.292	
	C	1.94 ± 0.42	1.87 ± 0.34	1.90 ± 0.29	0.249	0.780	0.005
CORE F_Close_	Dm	3.11 ± 0.59	2.39 ± 0.67	2.62 ± 0.54	5.925	0.005	0.208
	C	2.95 ± 0.84	2.16 ± 0.60	2.65 ± 0.69	9.600	0.001	0.143
CORE F_Social_	Dm	2.04 ± 0.48	2.50 ± 0.75	1.94 ± 0.67	2.275	0.114	
	C	2.05 ± 0.50	2.16 ± 0.51	2.04 ± 0.39	0.575	0.564	
CORE F_General_	Dm	0.40 ± 0.44	1.21 ± 0.57	0.87 ± 0.68	7.389	0.002	0.247
	C	0.68 ± 0.62	1.28 ± 0.73	1.01 ± 0.66	5.293	0.007	0.104
CORE R	Dm	0.02 ± 0.07	0.76 ± 1.05	0.36 ± 0.49	7.042	0.002	0.238
	C	0.11 ± 0.22	0.34 ± 0.42	0.40 ± 0.61	3.284	0.042	0.067
CORE R_Self._	Dm	0.02 ± 0.10	0.79 ± 1.07	0.19 ± 0.50	6.422	0.004	0.222
	C	0.03 ± 0.10	0.35 ± 0.59	0.30 ± 0.59	3.381	0.038	0.069
CORE R_Others_	Dm	0.02 ± 0.10	0.71 ± 1.07	0.71 ± 0.61	9.670	0.000	0.301
	C	0.25 ± 0.57	0.30 ± 0.39	0.62 ± 0.82	3.139	0.048	0.065

HbA1C = Glycosylated hemoglobin; Dm = Diabetes; C=Control; W = Subjective well-being; P = Psychological Problems; F = Functioning; R = Risk; P.S. = Physical symptoms; R. Self = Risk to self-harm; R. Others = Risk to harm to others; ŋ^2^ = Magnitude of the ANOVA effect.

## Data Availability

Data supporting reported results can be provided by authors by requesting those.
